# Supplementary material for: Folding and Intrinsic Disorder of the Receptor Tyrosine Kinase KIT Insert Domain Seen by Conventional Molecular Dynamics Simulations
Source: Int J Mol Sci. 2021 Jul 9;22(14):7375. doi: 10.3390/ijms22147375 (PMC8307779; doi:10.3390/ijms22147375)
Supplement: Supplementary file 1 [file ijms-22-07375-s001.zip › ijms-1268710-supplementary.pdf]

Supplementary Material

Folding and Intrinsic Disorder of the Receptor Tyrosine Kinase KIT Insert Domain Seen by Conventional Molecular Dynamics Simulation

Julie Ledoux, Alain Trouvé and Luba Tchertanov

Figures

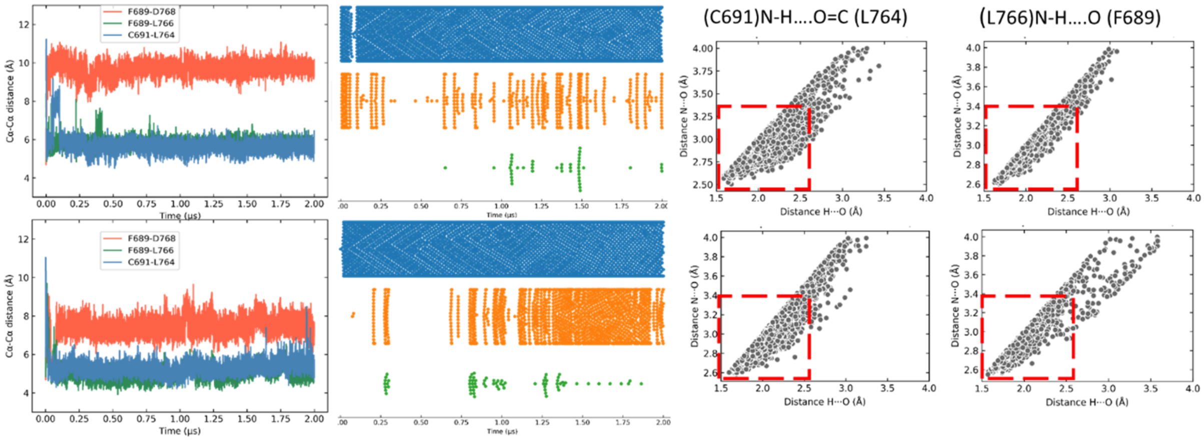

Figure S1. **Stabilising contacts between residues from N- and C- extremities of KID fused to KIT**. Distance between the pairs of Cα-Cα atoms (first columns at the left) and their occurrences over the MD simulations (second column). Distributions of distances between a donor (D) and an acceptor (A) and a hydrogen (H) and acceptor (A) characterising the strength of H-bonds (C691)N-H···O=C(L764) (third column) and (L766)N-H···O(F689) (fourth column).

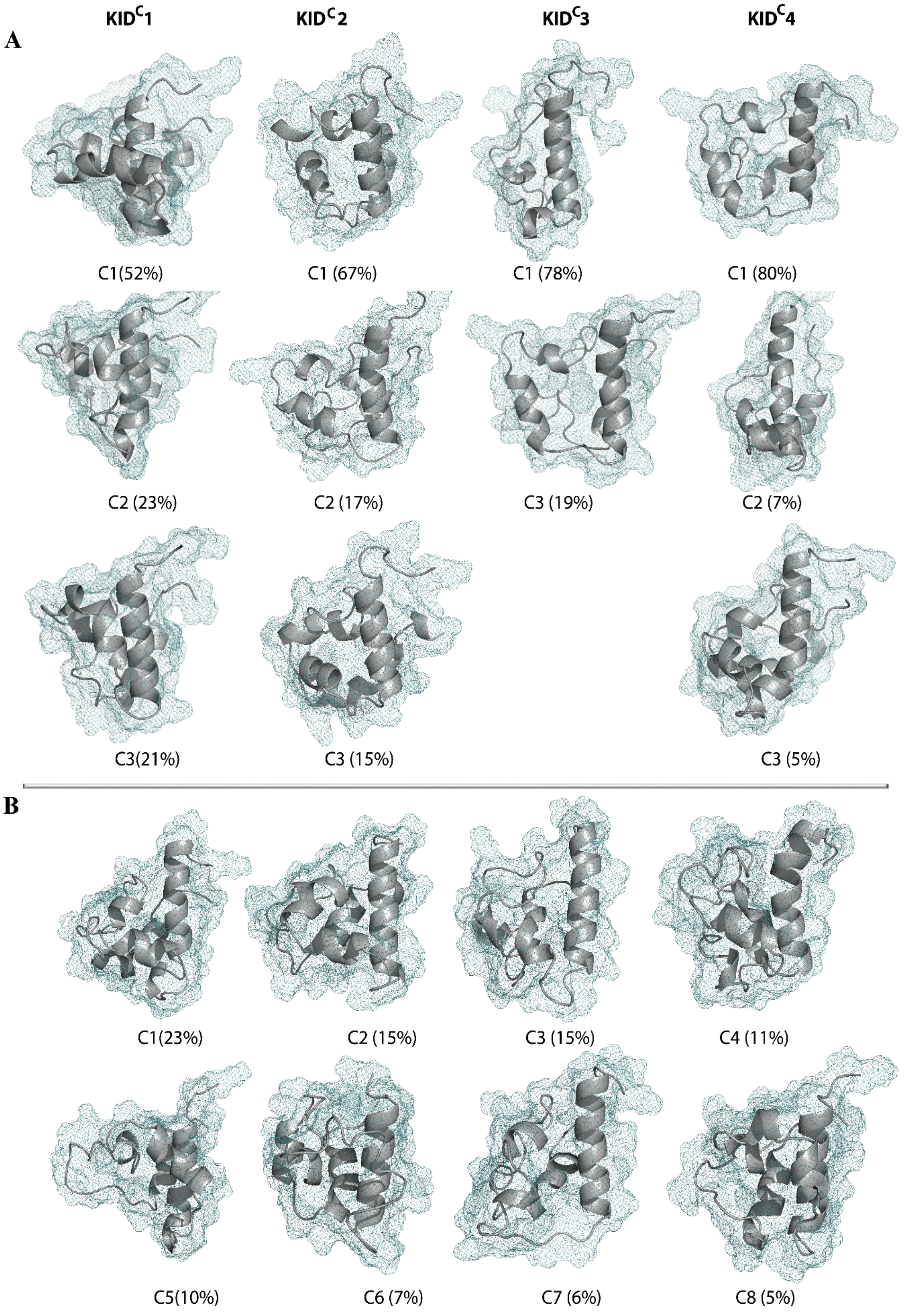

Figure S2. Clusters and their representative conformations of cleaved KID simulated as unconstrained species (KID^{C}). *Representative conformations* of clusters obtained on each trajectory (A) and concatenated trajectories (1-4) (B) of the MD simulation of KID. Conformations were clustered with the ensemble-based clustering using the RMSD cut-off of 4 Å. The first 100 ns as well as the most fluctuating residues from KID extremities with the RMSF values exceeding 6 Å were omitted from the analysis. KID is shown as a grey cartoon with a meshed surface. Population of each cluster is given in brackets.

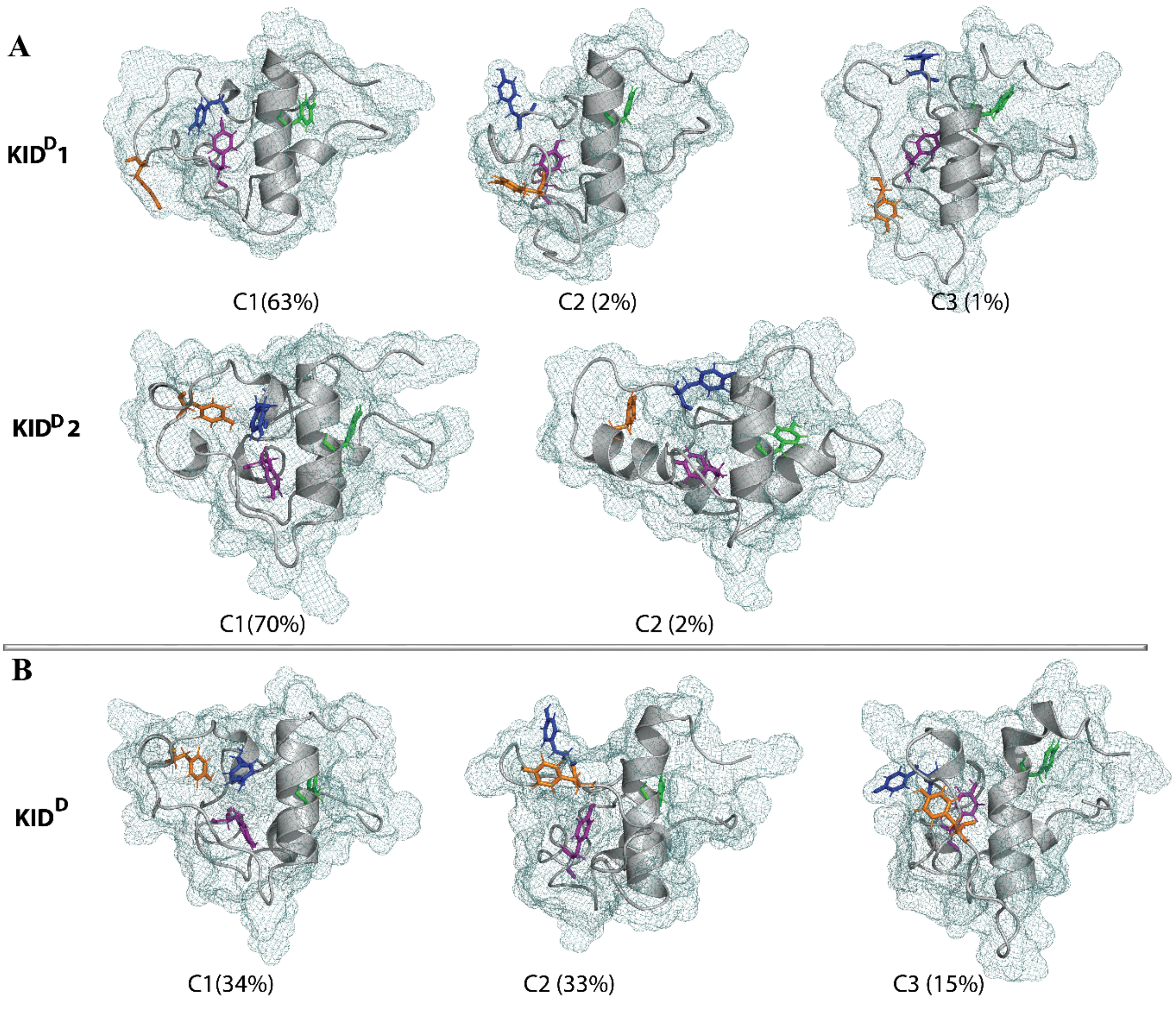

Figure S3. Clusters and their representative conformations of KID fused to KIT (KID^D). *Representative conformations* of clusters obtained on each trajectory **(A)** and concatenated trajectories (1-2) (B) of the MD simulation of KID. Conformations were clustered with the ensemble-based clustering using the RMSD cut-off of 4 Å. The first 100 ns as well as the most fluctuated residues from KID extremities with the RMSF values exceeding 6 Å were omitted from the analysis. KID is shown as a grey cartoon with the tyrosine residues (coloured differently) as sticks and a meshed surface. Population of each cluster is given in brackets.

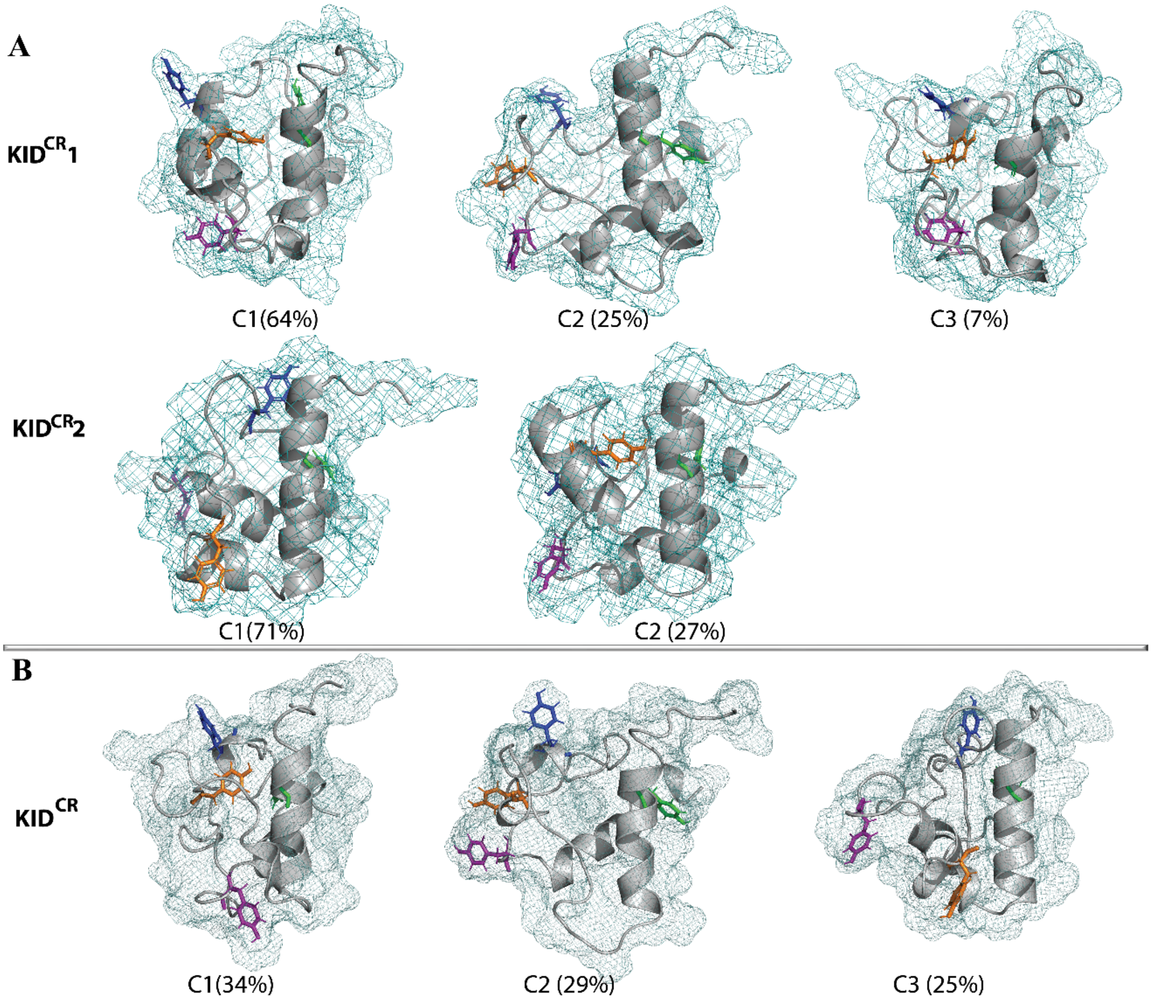

Figure S4. Clusters and their representative conformations of cleaved KID simulated with the constrained distance between its N- and C-ends (KID$^{\mathbf{CR}}$**).** *Representative conformations* of clusters obtained on each trajectory (A) and on concatenated trajectories (1-2) (B) of the MD simulation of KID. Conformations were clustered with the ensemble-based clustering using the RMSD cut-off of 4 Å. The first 100 ns as well as the most fluctuated residues from KID extremities with the RMSF values exceeding 6 Å were omitted from the analysis. KID is shown as a grey cartoon in grey with the tyrosine residues (coloured differently) as sticks and a meshed surface. Population of each cluster is given in brackets.

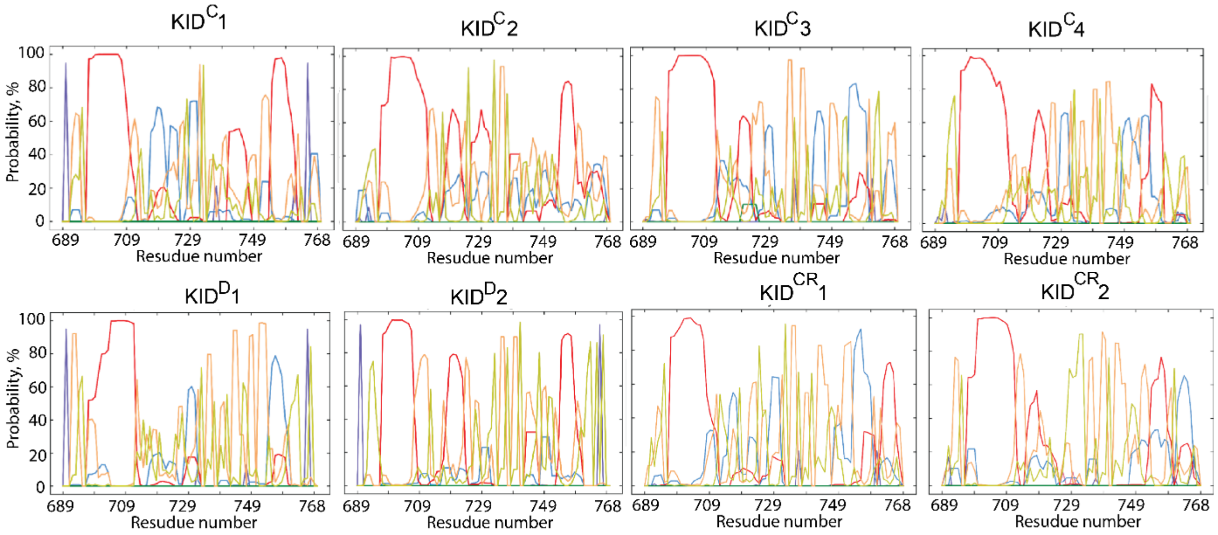

Figure S5. **Secondary structure assignment (by DSSP) of KID during MD simulations**. The proportion of every secondary structure type for each residue is given as a probability. Secondary structure is coded by colour: αH-, 3_{10}- and π-helices are in red, blue and green respectively; parallel and antiparallel strands are in rose and violet; turn and bend are in orange and pear.

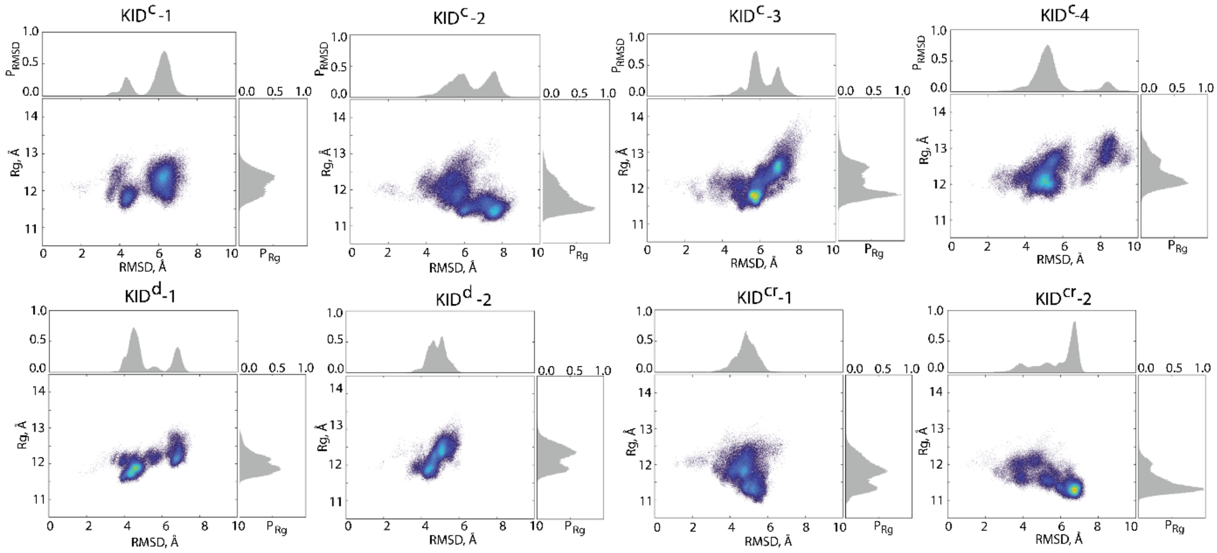

Figure S6. Compaction of KID estimated on conformational ensembles generated over each cMD trajectory for every KID entity studied. 2D representation of the free energy landscape of KID (KID^{c}, KID^{d} and KID^{cr}) conformational ensembles plotted as a function of Rg (in Å) versus RMSD (in Å). Probability distribution of R_g (right) and RMSD (top) calculated from each generated ensemble of KID studied as cleaved entity and as domain of KID. All calculations are performed on the Ca-atoms. The red colour represents the high energy state, yellow and green low and blue represents the lowest stable state. The free energy surface was plotted using Matlab.

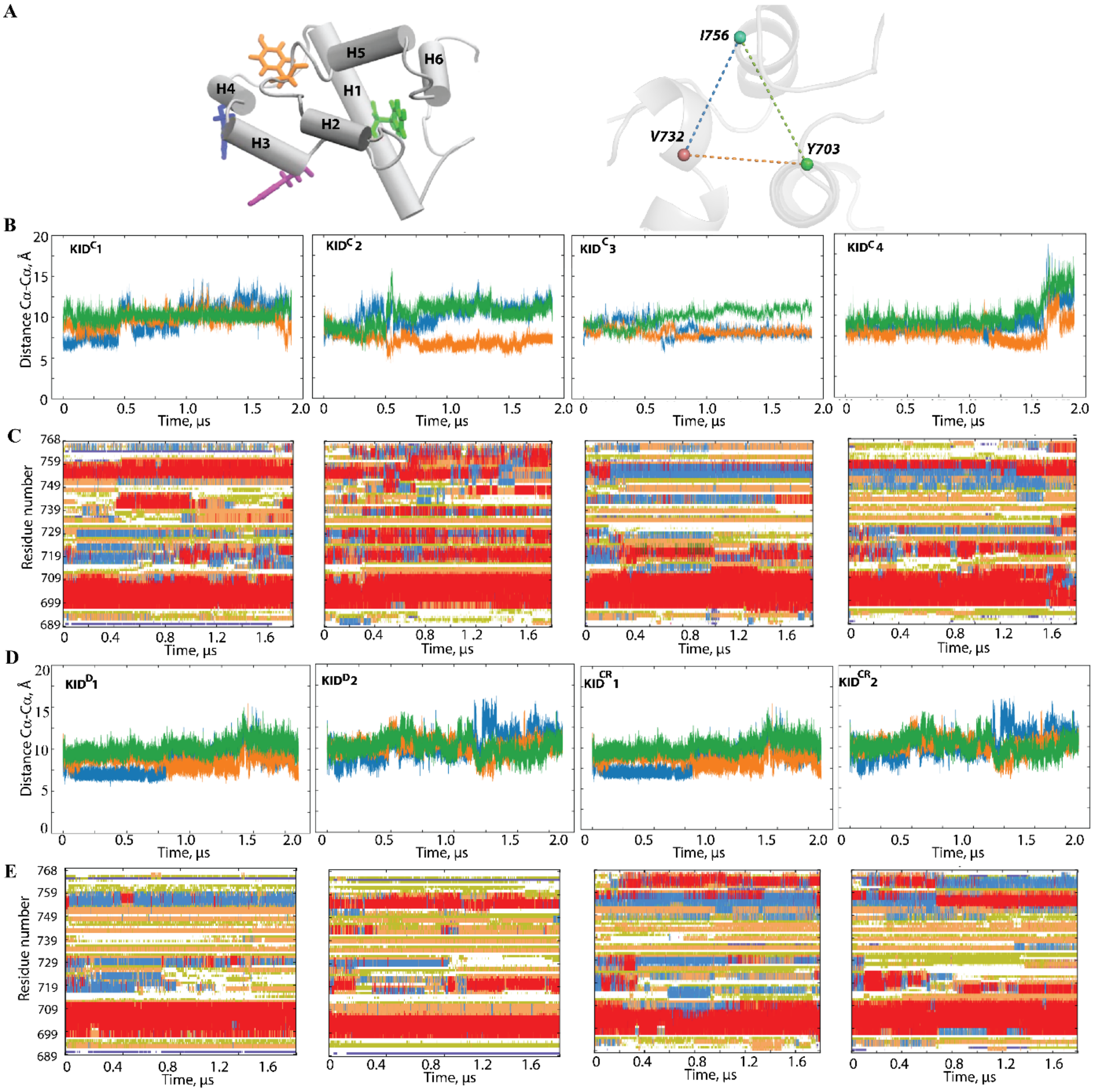

Figure S7. The inter-residue geometry of tyrosines in the isolated unconstrained KID and its relation with folding. (**A**) KID structure with helices shown as solid cylinders and tyrosine residues as sticks (left) and the triangle designed on the most stable over the MD simulations residues (with the smallest RMSF values) (right). (**B** and **D**) Distances between each pair of the tyrosine residues in each MD trajectory, coloured as the edges of triangle. (**C** and **E**) The time-related evolution of the secondary structures of each residue as assigned by DSSP with the type-coded secondary structure bar. (**D**) Distances between the most 'stable' (minimal RMSF values) residues Y703, V732 and I756, over each cMD trajectory, coloured as the edges of triangle in (**A**).

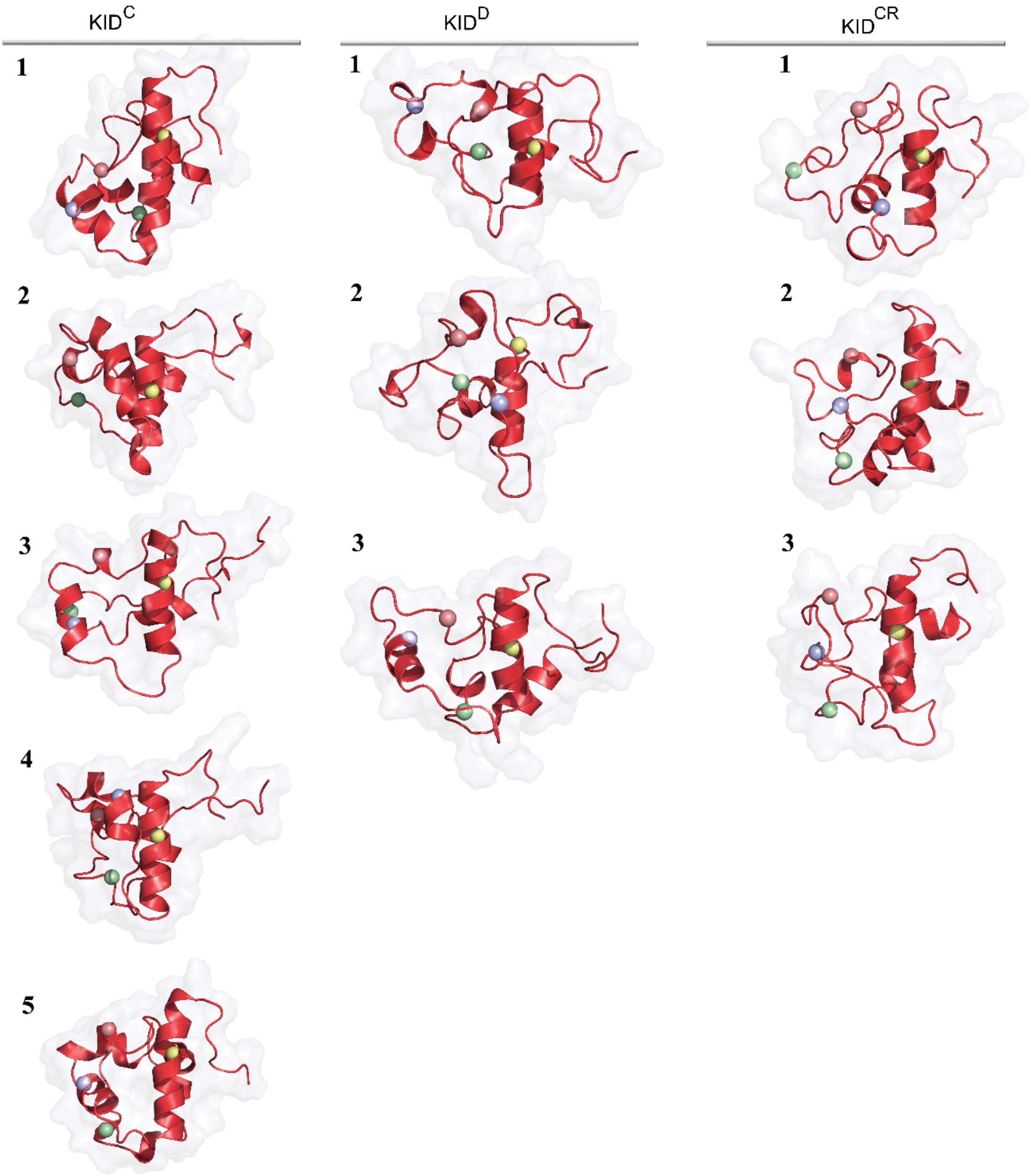

Figure S8. Representative conformations of KID from the deep wells on the free energy landscape (FEL) plotted as a function of *Rg versus* RMSD**.** KID is displayed as a red cartoon contoured with a surface-filled model. Position of the tyrosine residues (Cα-atoms) are shown as balls coloured in yellow, blue, red and green for Y703, Y721, Y730 and Y747 respectively.

Table S1. Comparison of the representative conformations of KID. The RMSD (Å) was calculated on the concatenated trajectories after least-square fitting.

1. Conformations taken from the deep wells found on FELs

KID	FEL	Well	Well	RMSD (Å)
KID^{C}	FEL_{RMSD}^{Rg}	1	2	8.4
			3	6.6
			4	7.0
			5	6.5
	FEL_{SASA}^{Rg}	1	2	4.8
KID^{D}	FEL_{RMSD}^{Rg}	1	2	7.1
			3	3.9
KID^{CR}	FEL_{RMSD}^{Rg}	1	2	5.8
			3	5.1
	FEL_{SASA}^{Rg}	1	2	5.2

2. Conformations taken from the deepest wells found on FELs

	FEL/FEL	RMSD (Å)
KID^{C}	FEL_{RMSD}^{Rg}/ FEL_{Hfp}^{Rg}	6.6
	FEL_{RMSD}^{Rg}/ FEL_{SASA}^{Rg}	6.7
	FEL_{Hfp}^{Rg} / FEL_{SASA}^{Rg}	1.9
KID^{D}	FEL_{RMSD}^{Rg}/ FEL_{Hfp}^{Rg}	2.0
	FEL_{RMSD}^{Rg}/ FEL_{SASA}^{Rg}	3.1
	FEL_{Hfp}^{Rg} / FEL_{SASA}^{Rg}	2.8
KID^{CR}	FEL_{RMSD}^{Rg}/ FEL_{Hfp}^{Rg}	6.9
	FEL_{RMSD}^{Rg}/ FEL_{SASA}^{Rg}	5.6
	FEL_{Hfp}^{Rg} / FEL_{SASA}^{Rg}	4.7

3. *Characterisation of conformational sub-sets from the deepest well on* FEL_{RMSD}^{Rg}

KID	Population, %	mv RMSD (Å)	mv Rg (A)
KID^{C}	11	5.70 (2)	11.80 (1)
KID^{CR}	19	6.69 (2)	11.33 (1)
KID^{D}	37	4.49 (1)	11.83 (1)

Note: The mean values are shown with the standard error.

4. *Characterisation of conformational sub-sets from the most populated cluster C1 (cut-off 4 Å) obtained by ensemble-based clustering*

KID	Population, %	mv RMSD (Å)	mv Rg (A)
KID^{C}	23	3 (1)	12.00 (3)
KID^{CR}	34	5 (1)	11.15 (4)
KID^{D}	34	3.9 (0.3)	11.83 (7)

Note: The mean values are shown with the standard error.

5. *Conformations taken from the most populated cluster C1 obtained by ensemble-based clustering*

KID	RMSD (Å)
KID^{C}/KID^{D}	6.54
KID^{C}/KID^{CR}	8.21
KID^{D}/KID^{CR}	7.03

6. *Conformations taken from the deepest wells found on FELs and from the most populated clusters obtained by the ensemble-based clustering*

KID	Cluster	FEL	RMSD (Å)
KID^{C}	C1	FEL_{RMSD}^{Rg}	7.9
	C1	FEL_{Hfp}^{Rg}	4.0
	C1	FEL_{SASA}^{Rg}	3.7
KID^{D}	C1	FEL_{RMSD}^{Rg}	4.2
	C1	FEL_{Hfp}^{Rg}	3.9
	C1	FEL_{SASA}^{Rg}	3.5
KID^{CR}	C1	FEL_{RMSD}^{Rg}	3.4
	C1	FEL_{Hfp}^{Rg}	3.8
	C1	FEL_{SASA}^{Rg}	2.6
